# Supplementary material for: AI-enabled, implantable, multichannel wireless telemetry for photodynamic therapy
Source: Nat Commun. 2022 Apr 21;13:2178. doi: 10.1038/s41467-022-29878-1 (PMC9023557; doi:10.1038/s41467-022-29878-1)
Supplement: Supplementary file 1 — Supplementary Information [file 41467_2022_29878_MOESM1_ESM.pdf]

# Supplementary Information

**Supplementary Figure 1.** A representative image of a user interface software.

**Supplementary Figure 2.** Measurements of temperature changes as a function of operation duty-cycle in wet and dry conditions.

**Supplementary Figure 3.** Summary of simulation results for Hypericin treatment.

**Supplementary Figure 4.** Summary of simulation results for Foscan treatment.

**Supplementary Figure 5.** Illustrative representations of $\boldsymbol{v}$ (an implantable device) and $\boldsymbol{a}_1$ & $\boldsymbol{a}_2$(Ant. # 1 & 2).

**Supplementary Figure 6.** Simulation results of discharging time at various conditions.

**Supplementary Figure 7.** Measurement results of light intensity during discharging.

**Supplementary Figure 8.** Device layout and a table of components used for an implantable wireless device.

**Supplementary Figure 9.** Signal-flows of an implantable device for multi-wavelength operation.

**Supplementary Figure 10.** Images of H&E stained sections of livers from mice in the different treatment groups.

**Supplementary Figure 11.** Tumor volumes were recorded on Days 0, 2, 4, and 5.

**Supplementary Figure 12.** Two representative images processed by the DeepLabCut-algorithm.

**Supplementary Figure 13.** Simulation results of a vertical structured antenna at various angles.

**Supplementary Figure 14.** Step-by-step procedures for device implantation.

# Supplementary Figure 1.

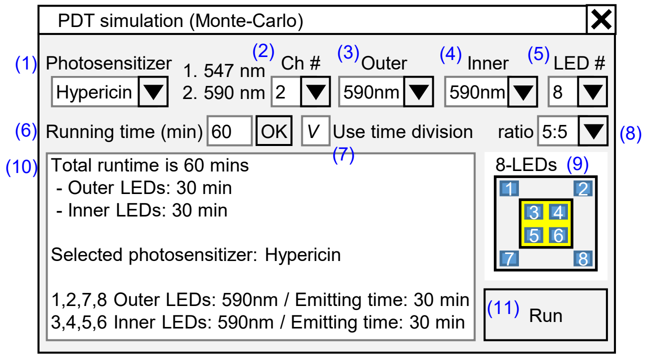


| | Setup value | Description |
|---|---|---|
| **1** | Photosensitizer type | Select the photosensitizer type; this version supports Foscan or Hypericin information. |
| **2** | The number of channels | Select how many channels use in the simulation. |
| **3** | Outer LED spectrum | Choose the spectrum of the outer LED, the location depends on the (5), the total number of LEDs |
| **4** | Inner LED spectrum | Choose the spectrum of the inner LED, the location depends on the (5), the total number of LEDs |
| **5** | The total number of LEDs | Select the total number of LEDs in a device. |
| **6** | Total runtime | Input the total running time of the simulation. |
| **7** | Time division function usage | Check whether to use the time division function or not. |
| **8** | The ratio of usage of outer and inner LED | If the user checked on (7), select the ratio of time division of LED usages. |
| **9** | Show the design of LEDs | Depends on the (5), it shows the design of the device |
| **10** | Description of setup values | All setup values are shown here. |
| **11** | Start button | If the setting values are okay, run the simulation.<br>If not, it shows an error message. |

**Supplementary Figure 1.** A representative image of a user interface software and detailed descriptions of parameter settings.

# Supplementary Figure 2.

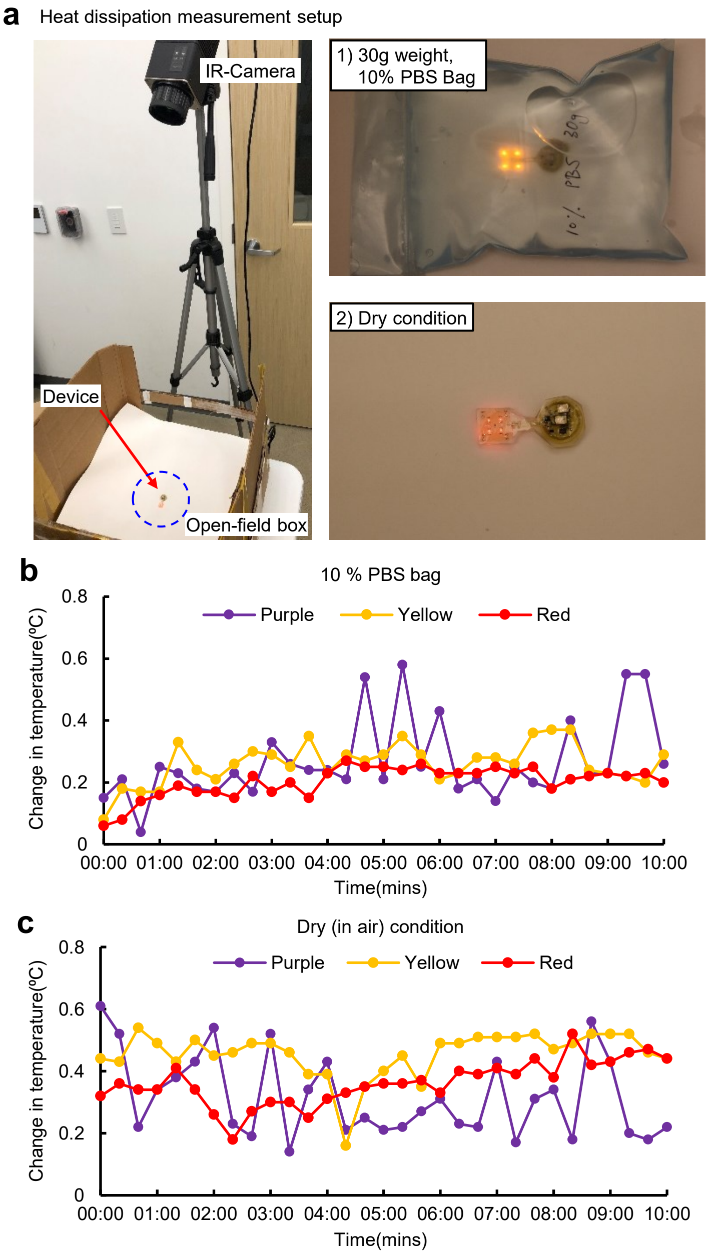


**Supplementary Figure 2.** (**a**) Pictures of an experimental setup for wireless measurements of heat dissipation using IR camera (left). Here, TX power is set to 4 W. The two right images show a device mounted on sealed bag of 10 % PBS saline solution (right to the top), and itself in a cage (right to the bottom), respectively. Plots of optical intensity as a function of time at duty cycles - 25 % with 10-ms pulse train - in each condition; 10 % PBS bag (**b**), and dry (**c**).

# Supplementary Figure 3.

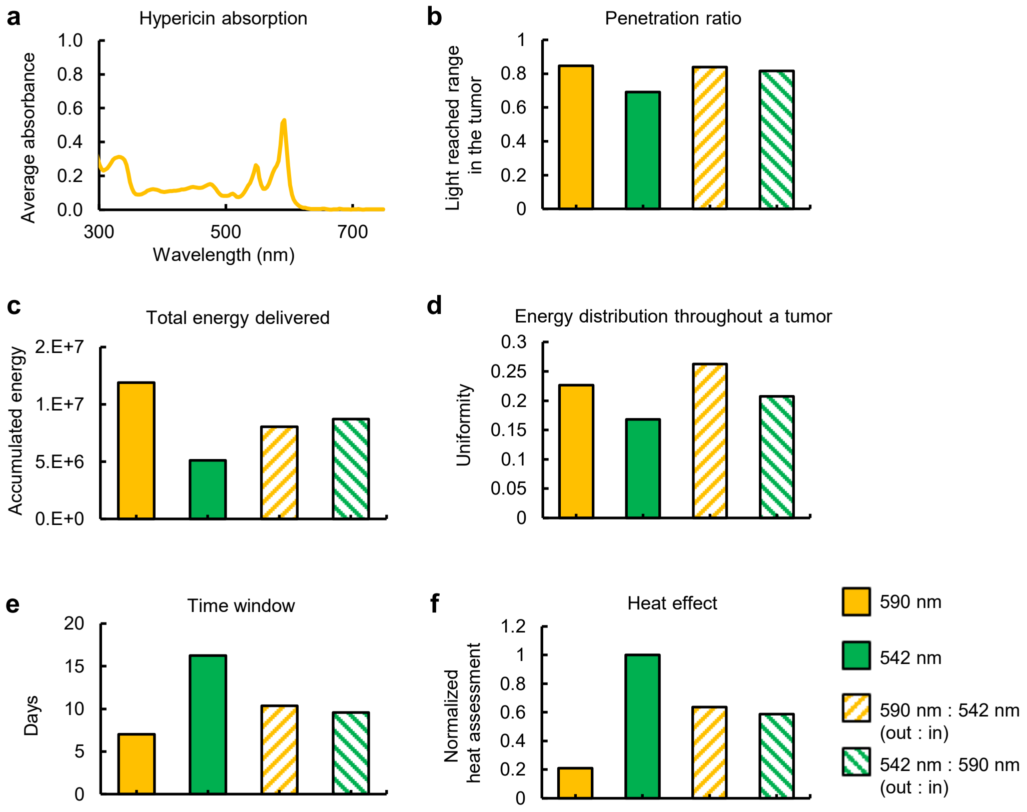


**Supplementary Figure 3.** Summary of simulation results for Hypericin treatment. (**a**) Averaged light absorbance spectrum of Hypericin: Peaks are located at 590 nm and 542 nm, respectively. (**b**) The percentage of the light energy bins reached in a tumor at various wavelengths. (**c**) Total energy delivered to a tumor at various wavelengths. (**d**) Energy distribution through a tumor at various wavelengths. (**e**) Time window during which experiments occur. Here, a total energy of 12 J $cm^{-3}$ is required for hindering tumor growth. Duty-cycle of light sources and light intensity determine the time window. (**f**) Normalized heat variation at various wavelength conditions. Results suggest that the use of light sources with a wavelength of 590 nm leads to minimum heat dissipation and maximum light absorption.

# Supplementary Figure 4.

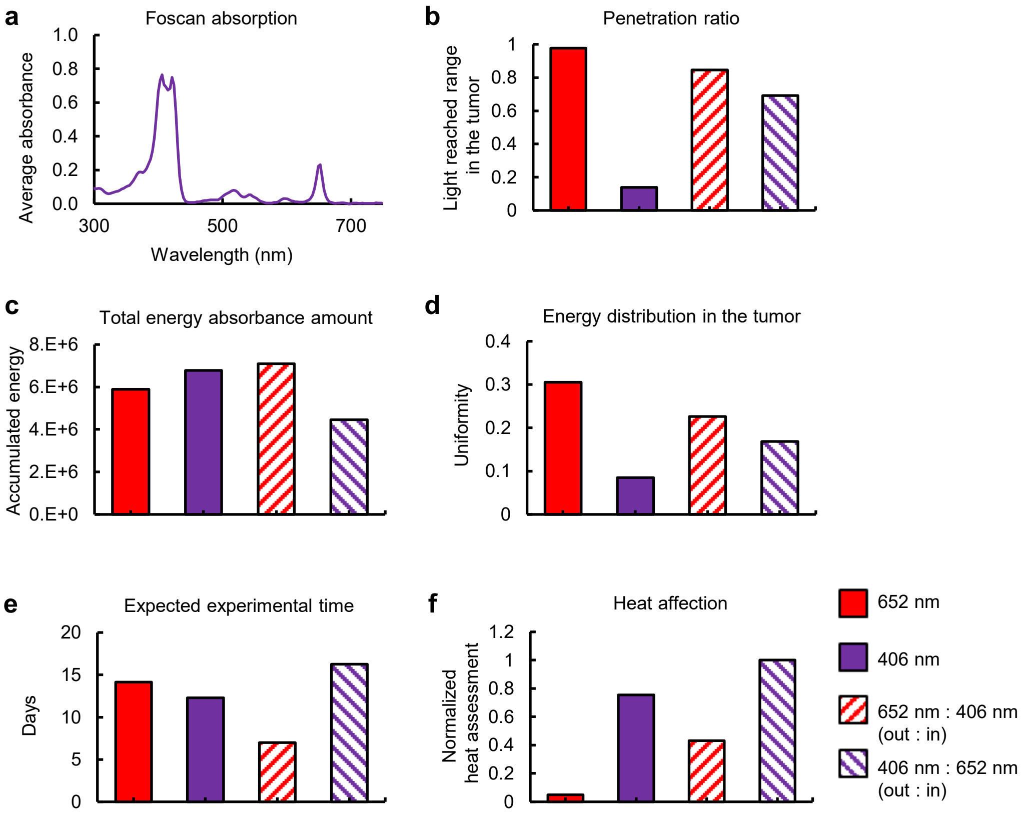


**Supplementary Figure 4.** Summary of simulation results for Foscan treatment. (**a**) Averaged light absorbance spectrum of Foscan: Peaks are located at 406 nm and 652 nm, respectively. (**b**) The percentage of the light energy bins reached in a tumor at various wavelengths. (**c**) Total energy delivered to a tumor at various wavelengths. (**d**) Energy distribution through a tumor at various wavelengths. (**e**) Time window during which experiments occur. Here, a total energy of 12 J $cm^{-3}$ is required for hindering tumor growth. Duty-cycle of light sources and light intensity determine the time window. (**f**) Normalized heat variation at various wavelength conditions. Results suggest that the use of light sources with a combination of wavelengths of 652 nm (out) and 406 nm (in) leads to minimum heat dissipation and maximum light absorption.

# Supplementary Figure 5.

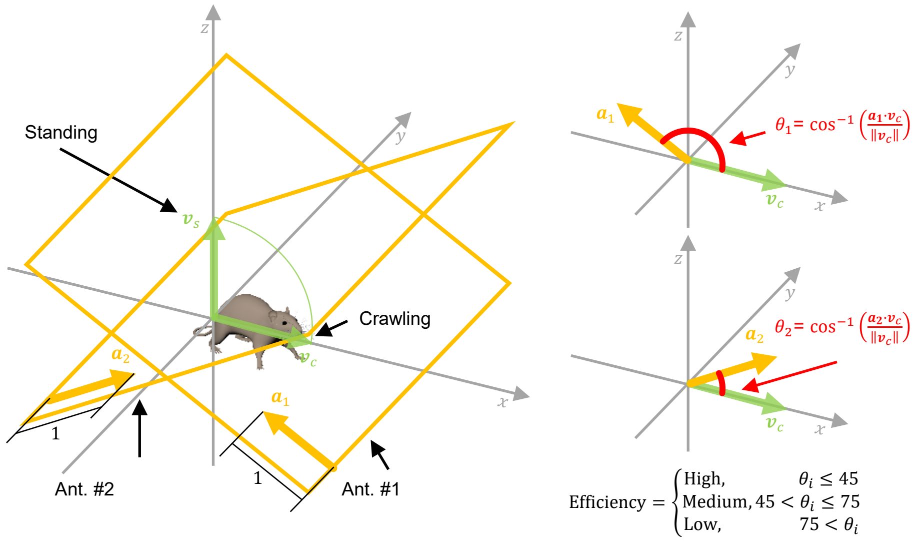


**Supplementary Figure 5.** Illustrative representations of $\boldsymbol{v}$ (an implantable device) and $\boldsymbol{a}_1$ & $\boldsymbol{a}_2$ (Ant. # 1 & 2). The relative angle $\theta_i$ between $\boldsymbol{v}$ and $\boldsymbol{a}_i$ determines the power transmission efficiency.

# Supplementary Figure 6.

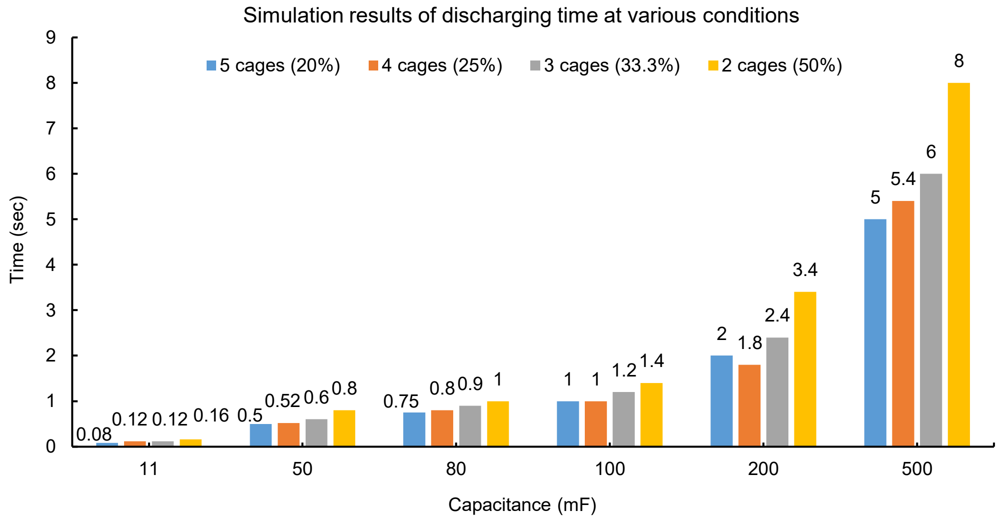


**Supplementary Figure 6.** Simulation results of discharging time at various conditions; different capacity and number of cages. Results suggest that a supercapacitor with a capacity of 11-mF can maintain light intensity enough for activation of a photosensitizer when not in powered by the TX system (during off-cycle).

# Supplementary Figure 7.

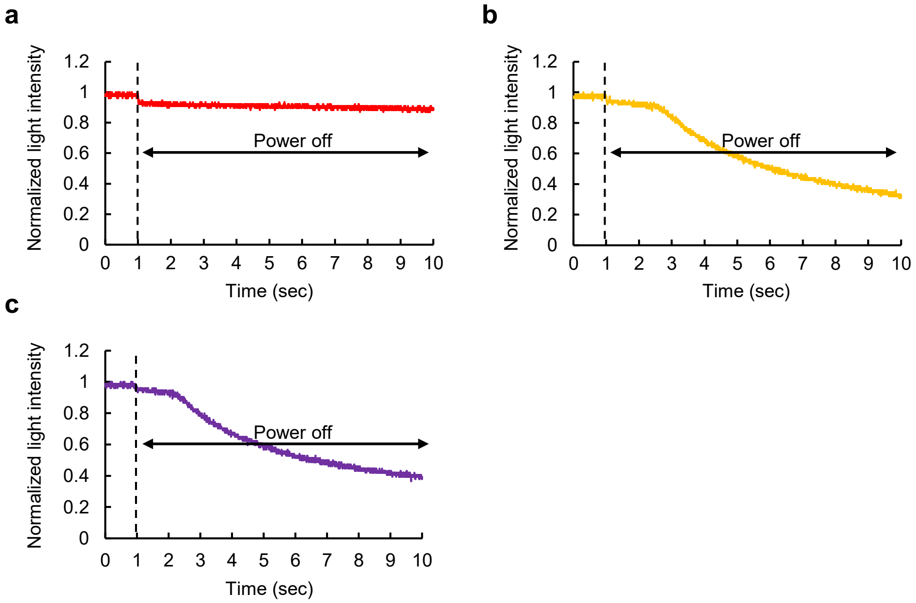


**Supplementary Figure 7.** Measurement results of light intensity during discharging. (**a**) red: 652 nm, (**b**) yellow: 590 nm, and (**c**) purple: 406 nm, respectively.

# Supplementary Figure 8.

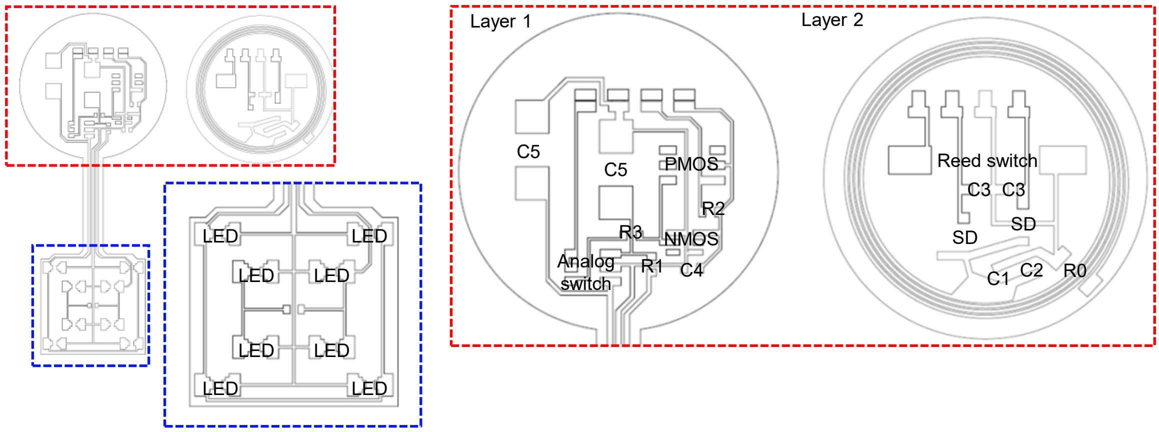


| | Components | Product number | Vendor information |
|---|---|---|---|
| **LED1** | 405nm, 1.6 mm x 0.8 mm x 0.8 mm | A-0603UUVC | Lighthouse LEDs |
| **LED2** | 591nm, 0.65 mm x 0.35 mm x 0.2 mm | APG0603SYC-TT | Kingbright |
| **LED3** | 632nm, 0.65 mm x 0.35 mm x 0.2 mm | APG0603SEC-E-TT | Kingbright |
| **SD** | Schottky Diode, 1.4 mm x 0.6 mm x 0.52 mm | DB27309 | Panasonic |
| **R0** | 0 ohm, 1.6 mm x 0.9 mm x 0.55 mm | RCWPM-0603 | VISHAY |
| **R1** | 0 ohm, 1.00 mm x 0.55 mm x 0.35 mm | RCWPM-0402 | VISHAY |
| **R2** | 20 kohm, 0.6 mm x 0.3 mm x 0.23 mm | RC0603F203CS | Samsung Electro-Mechanics |
| **R3** | 4.99 kohm, 0.6 mm x 0.3 mm x 0.23 mm | RC0603F4991CS | Samsung Electro-Mechanics |
| **C1** | 100 pF, 0.6 mm x 0.3 mm x 0.33 mm | CL03C820JA3NNNC | Samsung Electro-Mechanics |
| **C2** | 330 pF, 0.6 mm x 0.3 mm x 0.33 mm | CL03B331KA3NNNC | Samsung Electro-Mechanics |
| **C3** | 1 µF, 0.6 mm x 0.3 mm x 0.33 mm | CL03A105KP3NSNC | Samsung Electro-Mechanics |
| **C4** | 0.1 µF, 0.6 mm x 0.3 mm x 0.33 mm | CL03A104KP3NNNC | Samsung Electro-Mechanics |
| **C5** | 11 mF, 3.2 mm x 2.5 mm x 0.9 mm | CPH3225A | Seiko Instruments |
| **NMOS** | 1.0 mm x 1.0 mm x 0.34 mm | NTUD3170NZ | ON Semiconductor |
| **PMOS** | 1.7 mm x 1.7 mm x 0.6 mm | NX3008PBKV | Nexperia |
| **Reed Switch** | 7.0 mm x 2.2 mm x 1.6 mm | MK24-A-2 | Standex Meder electronics |
| **Analog Switch** | 1.6 mm x 1.2 mm x 0.6 mm | SN74LVC1G3157 | Texas Instruments |

**Supplementary Figure 8.** Device layout (top) and a table of components used for an implantable wireless device (bottom).

# Supplementary Figure 9.

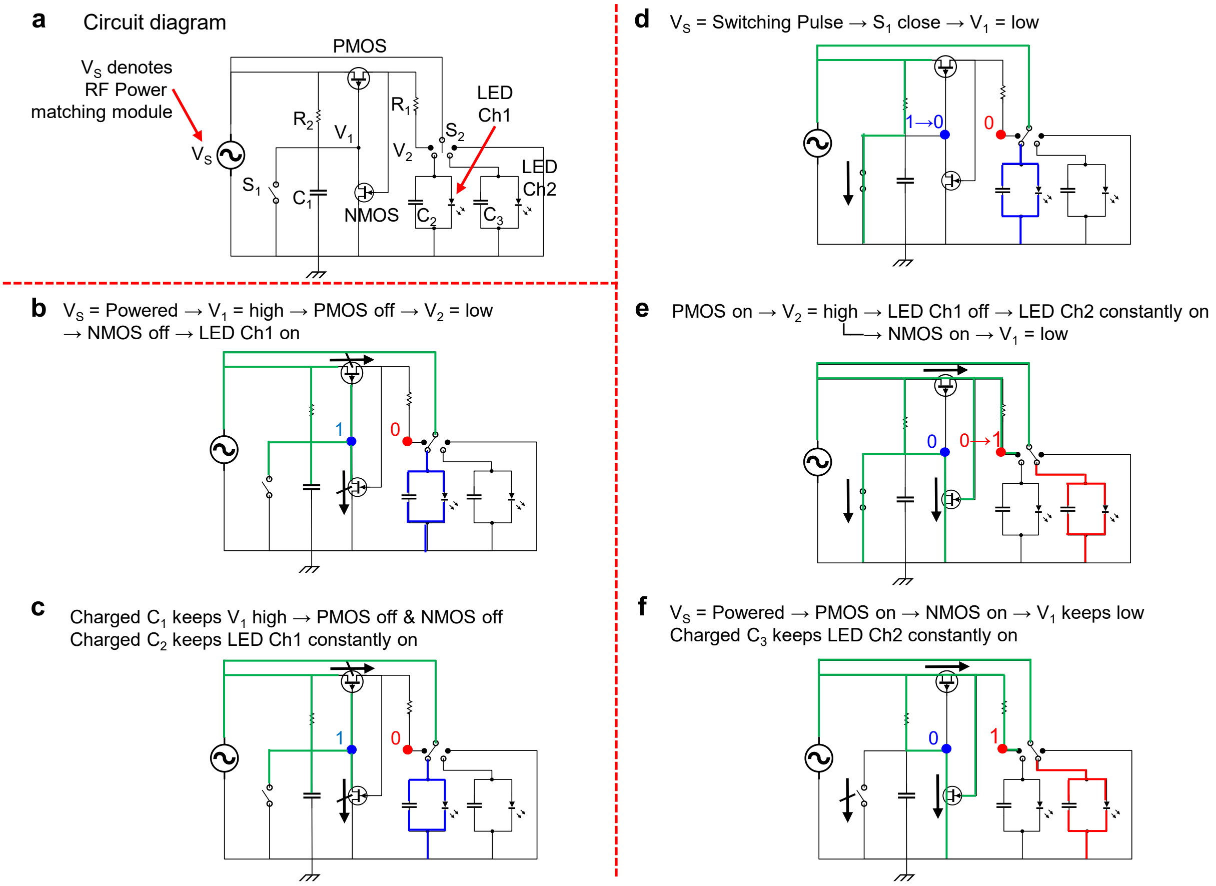


**Supplementary Figure 9.** (**a**) Circuit diagrams of an implantable device for multi-wavelength operation. Here, a dual-channel device automatically activates a channel in response to signals from a remotely located wireless TX system. $R_1 = 5\text{k}\Omega$, $R_2 = 20\text{k}\Omega$, $C_1 = 100\text{pF}$, $C_2 = C_3 = 11\text{mF}$, a reed switch is denoted by $S_1$, and $S_2$ denotes an analog switch. (**b**), (**c**) Signal-flows from a power supply to Ch1 LED for Ch1 activation. (**d**)-(**f**) Signal-flows during switching from Ch1 to Ch2.

# Supplementary Figure 10.

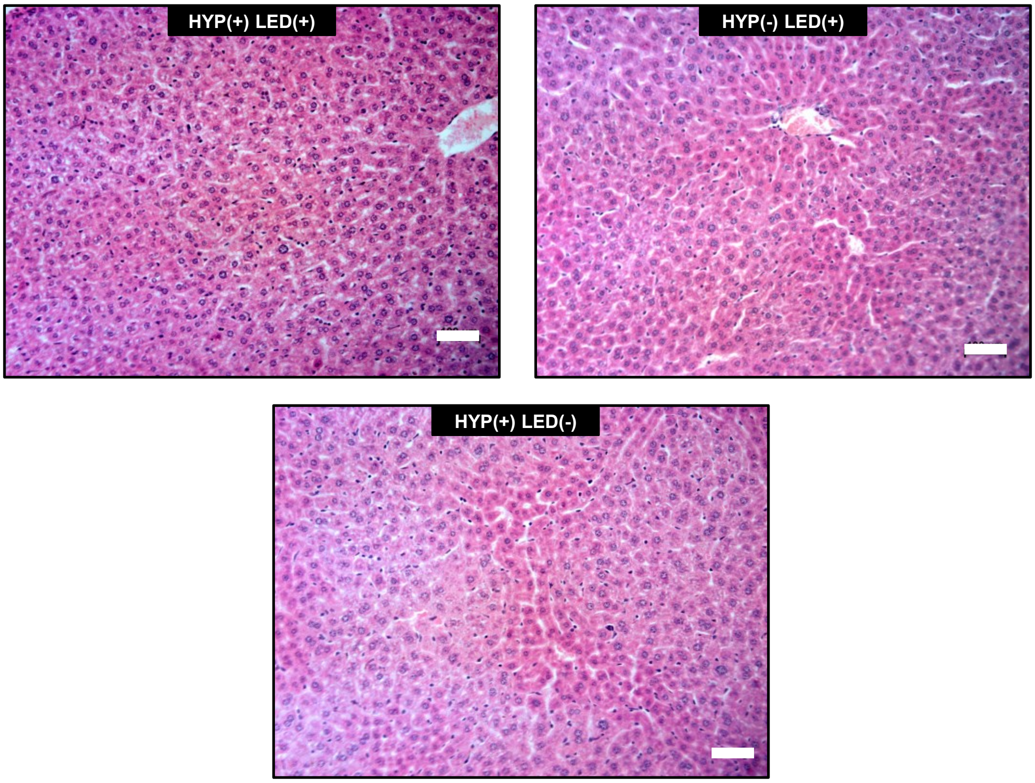


**Supplementary Figure 10.** Images of H&E stained sections of livers from mice in the different treatment groups: scale bar 100 µm. Images shown are representative examples of H&E staining performed on 3 independent livers per treatment group.

# Supplementary Figure 11.

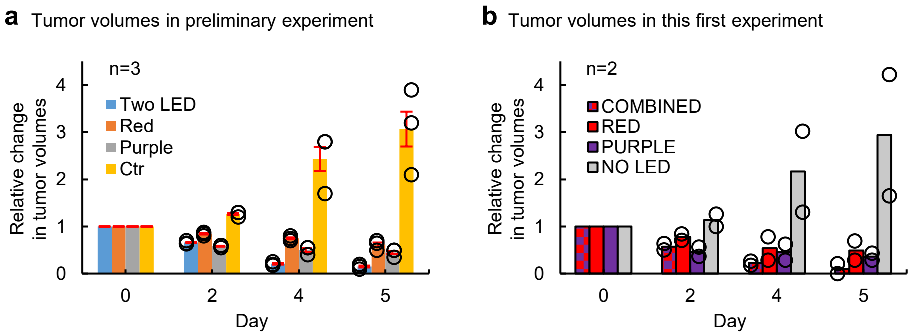


**Supplementary Figure 11.** Tumor volumes were recorded on Days 0, 2, 4, and 5. All groups received Foscan. The combined group received both red and purple LED light treatment. The red and purple LED light treatments only. No LED group received no LED light treatment. (**a**) Preliminary experiments and group size are 3 (n = 3). (**b**) The first experiments of this study and group size are 2 (n = 2). The only difference between two experiments is light dose. We used 0.45 J cm$^{-2}$ light fluency rate = 1.05 μW cm$^{-2}$) for (**a**) (left) while we did 0.43 J cm$^{-2}$ (light fluency rate = 1 μW cm$^{-2}$) for (**b**) (right); Data are presented as mean values +/- SD.

# Supplementary Figure 12.

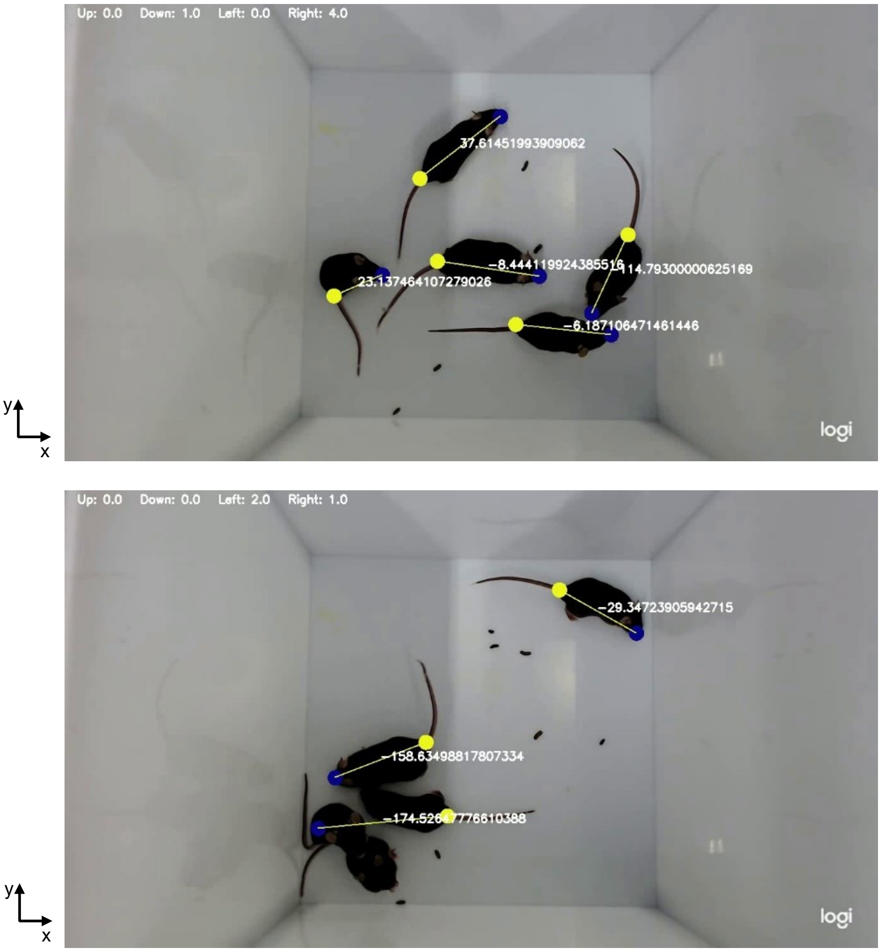


**Supplementary Figure 12.** Two representative images processed by the DeepLabCut algorithm. The top image shows perfect alignments of five vectors with a selected coil antenna while the bottom image includes only three vector assignments. It is likely for the two non-assigned mice (or implanted devices) to receive not enough power due to a misalignment between an implanted device and a selected coil antenna. However, a supercapacitor embedded in an implantable device can store power while harvesting energy from the TX system. Thus, it can still illuminate light sources when power delivery is not enough. Measurement results in supplementary Fig. 7 support this expectation.

# Supplementary Figure 13.

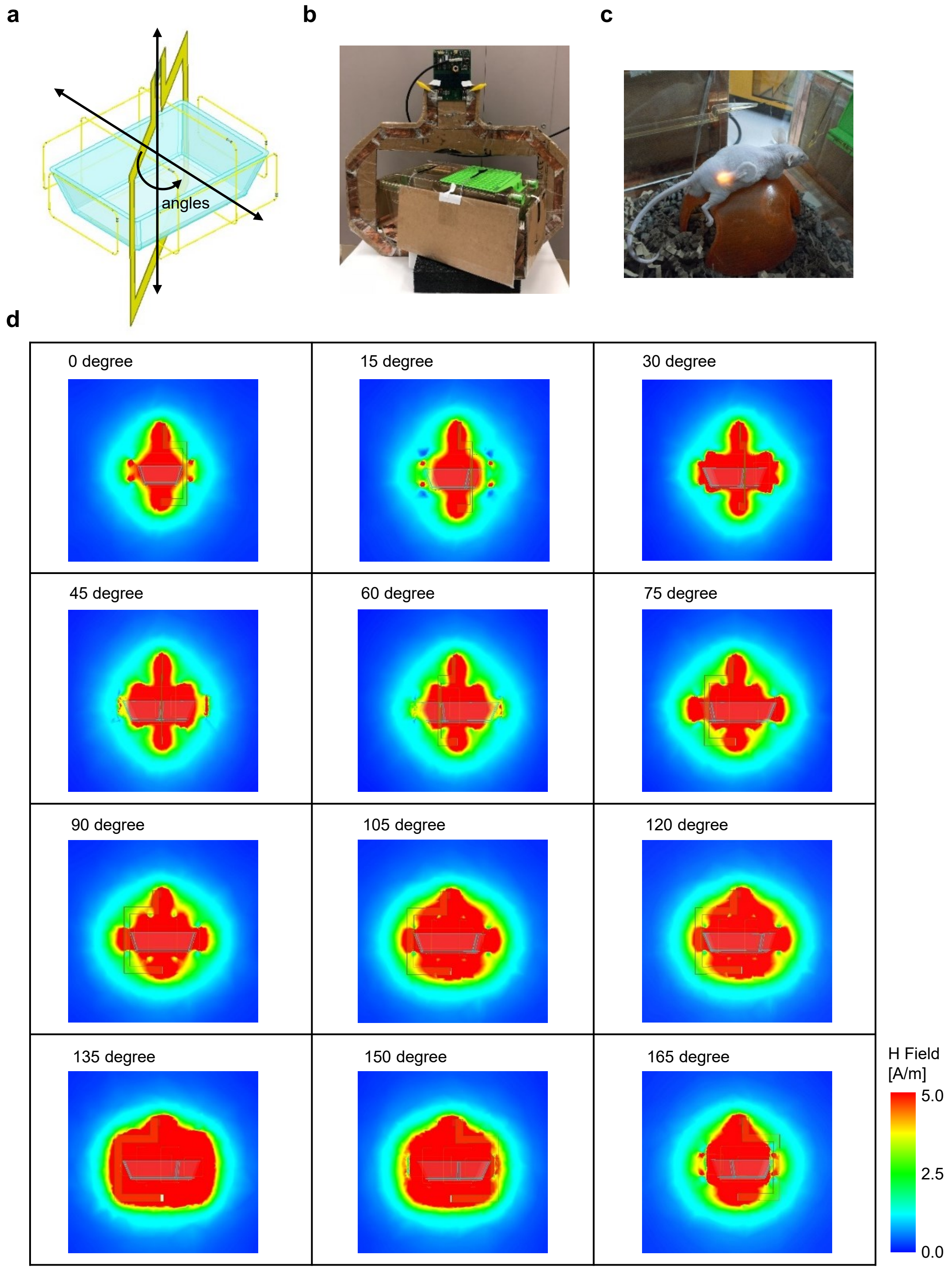


**Supplementary Figure 13.** (**a**) Illustration of the vertical structured antennas for flank implantation in a mouse. (**b**) A photo of the antenna setup and cage. (**c**) A photo of an animal with a device implanted. (**d**) Simulation results of a vertical structured antenna at various angles. Results reveal that the proposed structure offers uniform wireless coverage enough for activation of a photosensitizer throughout the volume of a cage.

# Supplementary Figure 14.

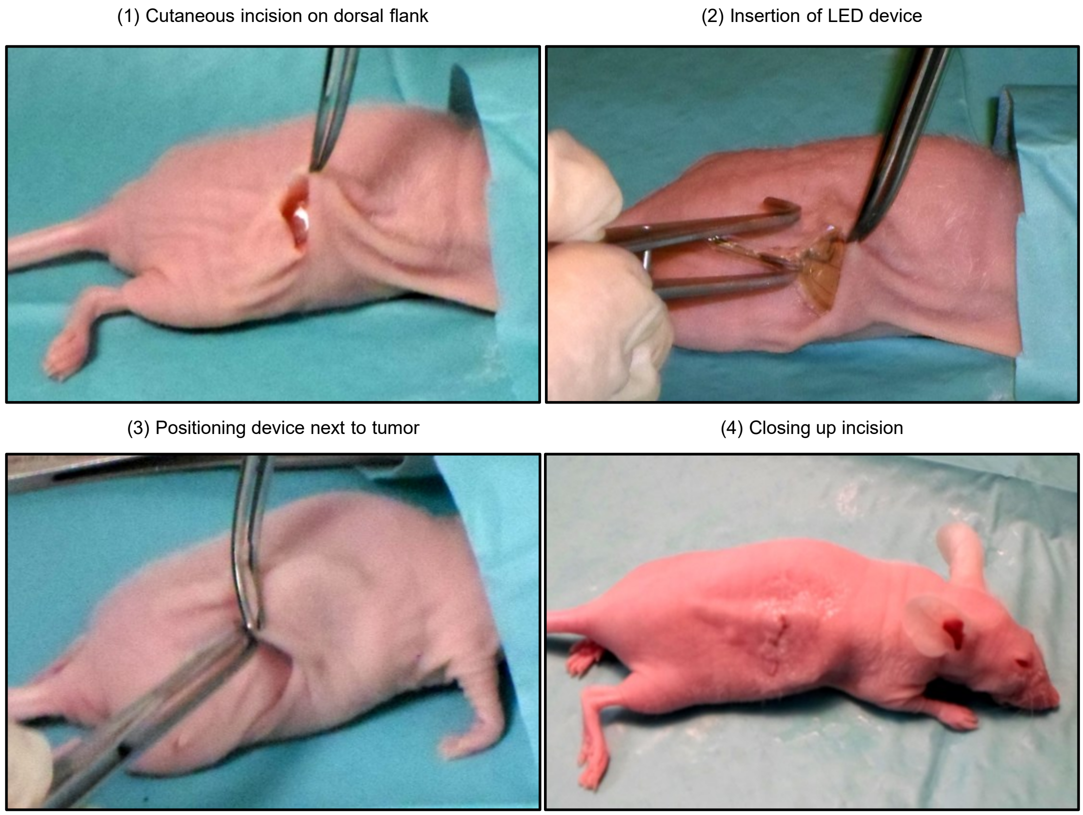


**Supplementary Figure 14.** Step-by-step procedures for device implantation.

# Supplementary Table 1

**Supplementary Table 1.** Summary of resulting python package information

| Package name | Version | Package name | Version | Package name | Version | Package name | Version |
|---|---|---|---|---|---|---|---|
| absl-py | 0.9.0 | msgpack | 1.0.0 | imgaug | 0.4.0 | qt | 5.9.7 |
| argon2-cffi | 20.1.0 | msgpack-numpy | 0.4.6.1 | importlib-metadata | 1.7.0 | qtconsole | 4.7.5 |
| astor | 0.8.1 | nb_conda | 2.2.1 | importlib_metadata | 1.7.0 | qtpy | 1.9.0 |
| astroid | 2.4.2 | nb_conda_kernels | 2.2.3 | intel-openmp | 2020.0.133 | readline | 8 |
| attrs | 19.3.0 | nbconvert | 5.6.1 | ipykernel | 5.3.4 | requests | 2.24.0 |
| backcall | 0.2.0 | nbformat | 5.0.7 | ipython | 7.17.0 | ruamel-yaml | 0.16.10 |
| bayesian-optimization | 1.2.0 | ncurses | 6.2 | ipython_genutils | 0.2.0 | ruamel-yaml-clib | 0.2.0 |
| blas | 1 | networkx | 2.4 | ipywidgets | 7.5.1 | scikit-image | 0.17.2 |
| bleach | 3.1.5 | notebook | 6.1.1 | isort | 5.4.2 | scikit-learn | 0.23.2 |
| c-ares | 1.15.0 | numba | 0.51.0 | jdcal | 1.4.1 | scipy | 1.5.2 |
| ca-certificates | 2020.7.22 | numexpr | 2.7.1 | jedi | 0.17.2 | send2trash | 1.5.0 |
| cairo | 1.14.12 | numpy | 1.16.4 | jinja2 | 2.11.2 | setuptools | 49.6.0 |
| certifi | 2020.6.20 | numpy-base | 1.19.1 | joblib | 0.16.0 | shapely | 1.7.0 |
| cffi | 1.14.1 | opencv-python | 4.1.2.30 | jpeg | 9b | sip | 4.19.8 |
| chardet | 3.0.4 | openpyxl | 3.0.5 | jsonschema | 3.2.0 | six | 1.15.0 |
| click | 7.1.2 | openssl | 1.1.1g | jupyter | 1.0.0 | sqlite | 3.33.0 |
| cudatoolkit | 10.0.130 | packaging | 20.4 | jupyter_client | 6.1.6 | statsmodels | 0.11.1 |
| cudnn | 7.6.5 | pandas | 1.1.1 | jupyter_console | 6.1.0 | tables | 3.6.1 |
| cupti | 10.0.130 | pandoc | 2.10.1 | jupyter_core | 4.6.3 | tabulate | 0.8.7 |
| cycler | 0.10.0 | pandocfilters | 1.4.2 | keras-applications | 1.0.8 | tensorboard | 1.13.1 |
| cython | 0.29.21 | pango | 1.45.3 | keras-preprocessing | 1.1.0 | tensorflow | 1.13.1 |
| dbus | 1.13.16 | parso | 0.7.0 | kiwisolver | 1.2.0 | tensorflow-base | 1.13.1 |
| decorator | 4.4.2 | patsy | 0.5.1 | lazy-object-proxy | 1.4.3 | tensorflow-estimator | 1.13.0 |
| deeplabcut | 2.2b7 | pcre | 8.44 | ld_impl_linux-64 | 2.33.1 | tensorflow-gpu | 1.13.1 |
| defusedxml | 0.6.0 | pexpect | 4.8.0 | libedit | 3.1.20191231 | tensorpack | 0.10.1 |
| easydict | 1.9 | pickleshare | 0.7.5 | libffi | 3.3 | termcolor | 1.1.0 |
| entrypoints | 0.3 | pillow | 7.2.0 | libgcc-ng | 9.1.0 | terminado | 0.8.3 |
| et-xmlfile | 1.0.1 | pip | 20.2.2 | libgfortran-ng | 7.3.0 | testpath | 0.4.4 |
| expat | 2.2.9 | pixman | 0.40.0 | libglu | 9.0.0 | threadpoolctl | 2.1.0 |
| filterpy | 1.4.5 | proglog | 0.1.9 | libpng | 1.6.37 | tifffile | 2020.8.13 |
| fontconfig | 2.13.0 | prometheus_client | 0.8.0 | libprotobuf | 3.13.0 | tk | 8.6.10 |
| freetype | 2.10.2 | prompt-toolkit | 3.0.5 | libsodium | 1.0.18 | toml | 0.10.1 |
| fribidi | 1.0.10 | prompt_toolkit | 3.0.5 | libstdcxx-ng | 9.1.0 | tornado | 6.0.4 |
| gast | 0.4.0 | protobuf | 3.13.0 | libuuid | 1.0.3 | tqdm | 4.48.2 |
| geos | 3.8.0 | psutil | 5.7.2 | libxcb | 1.14 | traitlets | 4.3.3 |
| gettext | 0.19.8.1 | ptyprocess | 0.6.0 | libxml2 | 2.9.10 | typed-ast | 1.4.1 |
| glib | 2.65.0 | pycparser | 2.2 | llvmlite | 0.34.0 | urllib3 | 1.25.10 |
| graphite2 | 1.3.14 | pygments | 2.6.1 | markdown | 3.2.2 | wcwidth | 0.2.5 |
| grpcio | 1.31.0 | pylint | 2.6.0 | markupsafe | 1.1.1 | webencodings | 0.5.1 |
| gst-plugins-base | 1.14.0 | pyparsing | 2.4.7 | matplotlib | 3.0.3 | werkzeug | 1.0.1 |
| gstreamer | 1.14.0 | pyqt | 5.9.2 | mccabe | 0.6.1 | wheel | 0.34.2 |
| h5py | 2.10.0 | pyrsistent | 0.16.0 | mistune | 0.8.4 | widgetsnbextension | 3.5.1 |
| harfbuzz | 2.4.0 | python | 3.7.7 | mkl | 2020.1 | wrapt | 1.11.2 |
| hdf5 | 1.10.6 | python-dateutil | 2.8.1 | mkl-service | 2.3.0 | wxpython | 4.0.4 |
| icu | 58.2 | pytz | 2020.1 | mkl_fft | 1.1.0 | xz | 5.2.5 |
| idna | 2.1 | pywavelets | 1.1.1 | mkl_random | 1.1.1 | zeromq | 4.3.2 |
| imageio | 2.9.0 | pyyaml | 5.3.1 | mock | 4.0.2 | zipp | 3.1.0 |
| imageio-ffmpeg | 0.4.2 | pyzmq | 19.0.1 | moviepy | 1.0.1 | zlib | 1.2.11 |
