# Supplementary material for: AI-enabled, implantable, multichannel wireless telemetry for photodynamic therapy
Source: Nat Commun. 2022 Apr 21;13:2178. doi: 10.1038/s41467-022-29878-1 (PMC9023557; doi:10.1038/s41467-022-29878-1)
Supplement: Supplementary file 2 — Reporting Summary [file 41467_2022_29878_MOESM2_ESM.pdf]

nature research

Corresponding author(s): Sung Il Park

Last updated by author(s): Mar 28, 2022

Reporting Summary

Nature Research wishes to improve the reproducibility of the work that we publish. This form provides structure for consistency and transparency in reporting. For further information on Nature Research policies, see our Editorial Policies and the Editorial Policy Checklist.

Statistics

For all statistical analyses, confirm that the following items are present in the figure legend, table legend, main text, or Methods section.

n/a	Confirmed	
☐	☒	The exact sample size (*n*) for each experimental group/condition, given as a discrete number and unit of measurement
☐	☒	A statement on whether measurements were taken from distinct samples or whether the same sample was measured repeatedly
☐	☒	The statistical test(s) used AND whether they are one- or two-sided<br>*Only common tests should be described solely by name; describe more complex techniques in the Methods section.*
☒	☐	A description of all covariates tested
☐	☒	A description of any assumptions or corrections, such as tests of normality and adjustment for multiple comparisons
☐	☒	A full description of the statistical parameters including central tendency (e.g. means) or other basic estimates (e.g. regression coefficient) AND variation (e.g. standard deviation) or associated estimates of uncertainty (e.g. confidence intervals)
☐	☒	For null hypothesis testing, the test statistic (e.g. *F*, *t*, *r*) with confidence intervals, effect sizes, degrees of freedom and *P* value noted<br>*Give P values as exact values whenever suitable.*
☒	☐	For Bayesian analysis, information on the choice of priors and Markov chain Monte Carlo settings
☒	☐	For hierarchical and complex designs, identification of the appropriate level for tests and full reporting of outcomes
☒	☐	Estimates of effect sizes (e.g. Cohen's *d*, Pearson's *r*), indicating how they were calculated

Our web collection on statistics for biologists contains articles on many of the points above.

Software and code

Policy information about availability of computer code

Data collection	For thermal assessment of devices, we used software IRBIS 3.1 plus (vesion 3.1.92).
Data analysis	We customized DLC Python package (V2.2b7: https://github.com/DeepLabCut/DeepLabCut) using Python 3.8.7 for the AI-enabled software of the proposed platform.<br>The integrated simulator used custom C code (Microsoft Visual Studio 2010 IDE) and script in MATLAB R2019b based on open source code (https://omlc.org/software/mc/mcxyz/index.html) and (https://doi.org/10.1016/j.celrep.2015.06.036).<br>Custom C code was used to program on the multiplexer controller (nRF52832 Development Kit, Nordic semiconductor). This code was based on C code libraries from Nordic (nRF5_SDK_13.0.0_04a0bfd) and Keil uVision 5 IDE(uVision V5.23.0.0).<br>For numerical electromagentic simulations, we used a finite element-method analysis tool (Ansys Electromagnetics Suite 17-HFSS, Ansys).<br>Statistics data were analyzed using Prism 9.0 (GraphPad Software).<br>The integrated simulator used custom C code (Microsoft Visual Studio 2010 IDE) and script in MATLAB R2019b based on open source code (https://omlc.org/software/mc/mcxyz/index.html) and (https://doi.org/10.1016/j.celrep.2015.06.036).<br>Custom C code was used to program on the multiplexer controller (nRF52832 Development Kit, Nordic semiconductor). This code was based on C code libraries from Nordic (nRF5_SDK_13.0.0_04a0bfd) and Keil uVision 5 IDE(uVision V5.23.0.0).<br>For numerical electromagentic simulations, we used a finite element-method analysis tool (Ansys Electromagnetics Suite 17-HFSS, Ansys).<br>Statistics data were analyzed using Prism 9.0 (GraphPad Software).<br>The code and the trained DLC model are available from GitHub (https://github.com/parkgroup-tamu/AI-enabled-implantable-multichannel-wireless-telemetry-for-photodynamic-therapy/tree/main/DeepLabCut_Modified).

For manuscripts utilizing custom algorithms or software that are central to the research but not yet described in published literature, software must be made available to editors and reviewers. We strongly encourage code deposition in a community repository (e.g. GitHub). See the Nature Research guidelines for submitting code & software for further information.

Data

Policy information about availability of data

All manuscripts must include a data availability statement. This statement should provide the following information, where applicable:

- Accession codes, unique identifiers, or web links for publicly available datasets
- A list of figures that have associated raw data
- A description of any restrictions on data availability

The data supporting the results in this study are available within the paper and its Supplementary Information. Source data are provided with this paper.

Field-specific reporting

Please select the one below that is the best fit for your research. If you are not sure, read the appropriate sections before making your selection.

[x] Life sciences [ ] Behavioural & social sciences [ ] Ecological, evolutionary & environmental sciences

For a reference copy of the document with all sections, see nature.com/documents/nr-reporting-summary-flat.pdf

Life sciences study design

All studies must disclose on these points even when the disclosure is negative.

Sample size	In vitro experiments - 3 independent experiments were performed and each experiment was performed in triplicates (3 data points). For the animal experiment<br>In vivo Hypericin experiment - 9 mice were used and 3 mice were placed into the 3 different treatment groups [HYP(-)LED(+), HYP(+)LED(-) and HYP(+)LED(+)].<br>In vivo Foscan experiment - 20 mice were used and 5 mice were placed into the 4 different treatment groups [COMBINED, RED, PURPLE, NO LED]<br>We (the authors) were in complete agreement about the number of experiments and sample sizes for each experiment to be adequate for portraying the significance of the results obtained in the experiments and also to satisfy requirements for statistical analyses.<br>No sample size calculation was used for either the in vitro cell line based or in vivo animal models experiments.<br>In vitro experiments were performed in triplicate per independent experiment to permit statistical analyses intra-experimentally and three independent experiments for statistical analyses inter-experimentally.<br>In vivo experiments were conducted to allow statistical significant findings to be obtained whilst reducing the number of animals per experiment. We found n=3 per treatment group for the Hypericin in vivo experiment to be adequate in obtaining significant findings and similarly for the Foscan in vivo experiment.
Data exclusions	No data was excluded
Replication	In vitro experiments were repeated independently 3 times with 3 readings (triplicate) per experiment. For the animal experiments, 3 and 5 mice were put into each different treatment group to confirm the reproducibility of results for the Hypericin and Foscan in vivo experiments respectively<br>All attempts at replication were successful.
Randomization	Randomization of HT29 cell cultures and animal subjects was not necessary in this study. HT29 cell cultures were identical for every experiment, introducing no variability. For in vivo experiments, the same Balb/c nude species, all female and all 6-8 weeks old were used and injected with the same number of HT29 cells, thereby reducing variability.
Blinding	Investigators were not blinded as it was not necessary in this study. All treatment groups were treated equally and only differentiated in their independent treatments.

Reporting for specific materials, systems and methods

We require information from authors about some types of materials, experimental systems and methods used in many studies. Here, indicate whether each material, system or method listed is relevant to your study. If you are not sure if a list item applies to your research, read the appropriate section before selecting a response.

Materials & experimental systems

n/a	Involved in the study
☒	☐ Antibodies
☐	☒ Eukaryotic cell lines
☒	☐ Palaeontology and archaeology
☐	☒ Animals and other organisms
☒	☐ Human research participants
☒	☐ Clinical data
☒	☐ Dual use research of concern

Methods

n/a	Involved in the study
☒	☐ ChIP-seq
☒	☐ Flow cytometry
☒	☐ MRI-based neuroimaging

Eukaryotic cell lines

Policy information about cell lines

Cell line source(s)	The human colorectal adenocarcinoma cell line, HT29, was obtained from the European Collection of Authenticated Cell Cultures (Salisbury, UK)
Authentication	HT29 obtained directly from European Collection of Authenticated Cell Cultures (Salisbury, UK). https://www.phe-culturecollections.org.uk/products/celllines/generalcell/detail.jsp?refId=91072201&collection=ecacc_gc. The cell line has been authenticated through genotyping.
Mycoplasma contamination	HT29 cell line is regularly tested for mycoplasma infection and tested negative before used for experiments.
Commonly misidentified lines (See ICLAC register)	No - HT29 is not listed as a Misidentified Cell Line on the ICLAC Register of Misidentified Cell Lines Database (Version 11 - Release Date: 8 Jun 2021)

Animals and other organisms

Policy information about studies involving animals; ARRIVE guidelines recommended for reporting animal research

Laboratory animals	Female BALB/c nude mice (6-8 weeks old). Mice were housed in the GM500 Mouse IVC Green Line cage (Tecniplast UK, London, UK) and kept on the Sealsafe Plus Mouse DGM cage rack (Tecniplast UK). Mice were kept on a 12-hour light and 12-hour dark cycle, at 25 °C room temperature and ambient room humidity.
Wild animals	Study did not involve wild animals
Field-collected samples	Study did not involve samples collected in the field
Ethics oversight	Animal experiment was performed at the University of Leeds under a personal project home office license held by P. Louise Coletta. The in vivo experiments were conducted within the Leeds Institute of Medical Research (University of Leeds, UK). Study was conducted in line with the Home Office regulations and in accordance with The Animals (Scientific Procedures) Act 1986, under a personal project animal license (License number: P93AOF172).

Note that full information on the approval of the study protocol must also be provided in the manuscript.
