# Supplementary material for: AI-enabled, implantable, multichannel wireless telemetry for photodynamic therapy
Source: Nat Commun. 2022 Apr 21;13:2178. doi: 10.1038/s41467-022-29878-1 (PMC9023557; doi:10.1038/s41467-022-29878-1)
Supplement: Supplementary file 4 — Description of Additional Supplementary Files [file 41467_2022_29878_MOESM4_ESM.pdf]

Title: Supplementary Movie 1.
Description: Demonstration of multi-cage activation using a single power source.

Title: Supplementary Movie 2.
Description: In vivo demonstration of activation of 5-mice in a cage.
